# Diverse Effects of Phytoestrogens on the Reproductive Performance: Cow as a Model

**DOI:** 10.1155/2013/650984

**Published:** 2013-04-23

**Authors:** Izabela Wocławek-Potocka, Chiara Mannelli, Dorota Boruszewska, Ilona Kowalczyk-Zieba, Tomasz Waśniewski, Dariusz J. Skarżyński

**Affiliations:** ^1^Department of Reproductive Immunology and Pathology, Institute of Animal Reproduction and Food Research, Polish Academy of Sciences, Tuwima 10 Street, 10-747 Olsztyn, Poland; ^2^Department of Life Sciences, Doctoral School in Life Sciences, University of Siena, Miniato via A. Moro 2 St., 53100 Siena, Italy; ^3^Department of Gynecology and Obstetrics, Faculty of Medical Sciences, University of Warmia and Masuria, Zolnierska 14 C St., 10-561 Olsztyn, Poland

## Abstract

Phytoestrogens, polyphenolic compounds derived from plants, are more and more common constituents of human and animal diets. In most of the cases, these chemicals are much less potent than endogenous estrogens but exert their biological effects via similar mechanisms of action. The most common source of phytoestrogen exposure to humans as well as ruminants is soybean-derived foods that are rich in the isoflavones genistein and daidzein being metabolized in the digestive tract to even more potent metabolites—*para*-ethyl-phenol and equol. Phytoestrogens have recently come into considerable interest due to the increasing information on their adverse effects in human and animal reproduction, increasing the number of people substituting animal proteins with plant-derived proteins. Finally, the soybean becomes the main source of protein in animal fodder because of an absolute prohibition of bone meal use for animal feeding in 1995 in Europe. The review describes how exposure of soybean-derived phytoestrogens can have adverse effects on reproductive performance in female adults.

## 1. **Introduction**

The present paper focuses particularly on soybean-derived isoflavones and summarizes recent knowledge on their biological impact on ruminant and human reproduction. Phytoestrogens belong to a heterogenous group of herbal substances with their structure similar to estradiol-17*β* (E_2_). They are called estrogen-like molecules or nonsteroidal estrogens structurally similar to E_2_. Phytoestrogens are diphenolic as well as nonsteroidal compounds.

**Figure 1:**
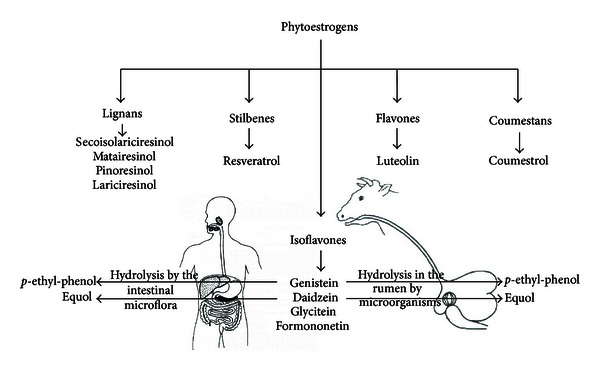
Classification and metabolism of phytoestrogens.

Systematically, the group of phytoestrogens includes over 100 molecules, divided according to their chemical structure into: isoflavones (genistein, daidzein, glycitein, and formononetin), flavones (luteolin), coumestans (coumestrol), stilbenes (resveratrol), and lignans (secoisolariciresinol, matairesinol, pinoresinol, and lariciresinol) [1] (Figure 1). Isoflavones are found at high concentrations in soybean products whereas lignans are found in flax seed, coumestans are found in clover, and stilbenes are found in cocoa- and grape-containing products, particularly red wine.

Phytoestrogens have recently come into considerable interest due to the following facts: first increasing information on their adverse effects in human and animal reproduction, second an increasing number of people substituting animal proteins with plant-derived proteins. Finally, the soybean becomes the main source of protein in animal (especially, dairy cows, pigs, and poultry species) fodder because of an absolute prohibition of bone meal use for animal feeding in 1995 in Europe.

There is some evidence that consumption of soy diets containing phytoestrogens has some positive effects on human and animal health. Phytoestrogens as potent antioxidants [2] are thought to reduce the risk of mammary cancer [3, 4], prevent cardiovascular disease [5], stop the progression of atherosclerosis [6], or have positive effects on hot flushes, vaginal symptoms, cognitive function, or dementia in postmenopausal women [7]. On the other hand, these substances also have some hazardous effects, especially in animals fed with pasture rich in phytoestrogens [8, 9]. The earliest evidence that naturally occurring phytoestrogens could cause reproductive disturbances in mammals was reported in 1946 by Bennetts et al. [10] indicating that ingestion of clover pasture rich in plant estrogens caused infertility in sheep. About 20 years later, a similar observations had been noted in cows that had fertility disturbances resulting from periods of feeding with red clover [11, 12]. Similarly, abnormalities in reproductive health due to high intake of soy products have been reported in women [13–16]. These observations demonstrate that dietary phytoestrogens can have adverse effects on reproductive performance in female adults.

## 2. **Mechanism of Isoflavone Action**

**Figure 2:**
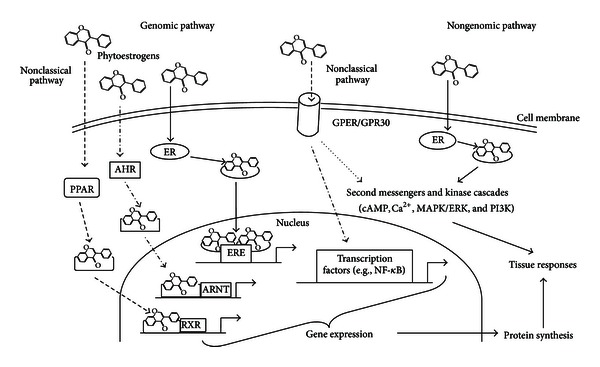
Schematic model illustrating the possible mechanisms of phytoestrogen action (the abbreviations on the figure stand for: AHR—aryl hydrocarbon receptor; ARNT—AHR nuclear translocator; ER—estrogen receptor; ERE—estrogen response element; cAMP—cyclic adenosine monophosphate; Ca^2+^—calcium ions; GPER/GPR30—G protein-coupled estrogen receptor 1; MAPK/ERK—mitogen-activated protein kinases/extracellular-signal-regulated kinases; NF-*κ*B—nuclear factor kappa-light-chain-enhancer of activated B cells; PI3 K—phosphatidylinositide 3-kinases; PPAR—peroxisome-proliferator-activated receptor; RXR—retinoid X receptor).

Environmental estrogens exert their effects through classical, genomic, or nongenomic pathways (Figure 2). Due to their similarity with the endogenous hormones, these compounds can bind to nuclear receptors. Their affinities for ER*α* and ER*β* are relatively weak compared to endogenous E_2_; thus, they can have agonist or antagonist activity depending on the presence of E_2_ [17]. It has been proved that some isoflavones are selective estrogen receptor modulators that have higher affinity to ER*β* than ER*α* [18, 19]. Environmental estrogens have much lower (up to 100 fold) affinity for nuclear receptors compared to the endogenous ligands (E_2_). Thus, even low concentrations of environmental estrogens can trigger an altered response of the biological systems. This interference is often achieved by the activation of nongenomic pathways. There are numerous nongenomic pathways affected by isoflavones, such as nongenomic signaling mediated by oxidative stress pathways, tyrosine kinases, nuclear factor-kappaB, and extracellular-signal-regulated kinases [20, 21]. In addition to classical ERs, isoflavones serve as ligands for peroxisome-proliferator-activated receptors, the nonclassical estrogen receptor GPER1, the estrogen-related receptors, and the aryl hydrocarbon receptor [20, 22–24]. Besides these direct actions to modulate signaling pathways, isoflavones can alter epigenetic marks by altering activities of DNA and histone methyltransferases, NAD-dependent histone deacetylases, and other modifiers of chromatin structure [25–27]. The last, described in the literature, way of isoflavone action in the cells is the competitive inhibition of the production of endogenous E_2_ by aromatase [27, 28]. The action of isoflavones in the human or animal body is even more complex since these substances are usually present *in vivo* as mixtures of several dietary components that can affect various signaling pathways or affect the same pathways in opposing directions.

## 3. **Adverse Effects of Isoflavones on the Reproductive Performance in Ruminants**

### 3.1. Metabolism and Bioavailability of Phytoestrogens

In the late 80s and early 90s, there were a lot of studies on feeding dairy cows with synthetic fodder containing phytoestrogens. The fodder commonly used for feeding dairy cattle contains phytoestrogens, such as genistein, daidzein, formonentin, and biochanin A [29]. Lundh et al. [30] showed that in cows and ewes daidzein and genistein present in the fodder are immediately converted in the rumen to equol and *p*-ethyl-phenol, respectively (Figure 1). The concentration of daidzein and genistein decreases within one hour after feeding, whereas equol and *p*-ethyl-phenol are present in the blood of cows for many hours after feeding [30]. The metabolism of phytoestrogens from synthetically prepared fodder, rich in phytoestrogens was thoroughly investigated by Lundh et al. [29, 30]. However, we were the first to study the effects of feeding cattle with fodder rich in phytoestrogens derived from natural soybean [31, 32]. At the beginning, we established which metabolites of phytoestrogens are present in the blood of cows fed a diet rich in soybean. We found large amounts of daidzein and genistein in the soybean commonly used for feeding dairy cattle [31]. These phytoestrogens occur in plants as glycosides and are hydrolysed in the rumen by microorganisms [33]. Daidzein is metabolized in the rumen to equol, whereas genistein is metabolized to *p*-ethyl-phenol [30, 33]. We found high concentrations of both of these metabolites in blood plasma and urine of the cows fed with high-soybean-based diet [31].

**Figure 3:**
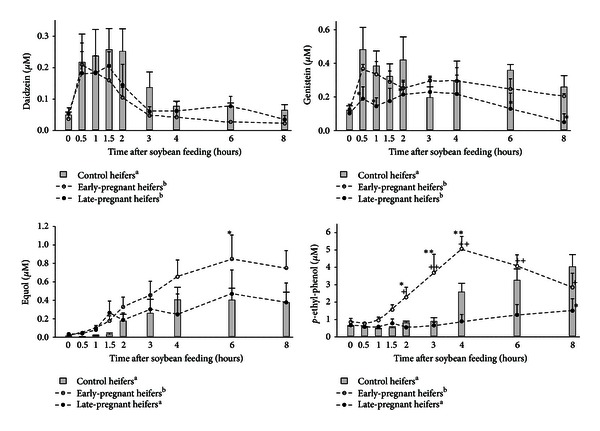
Time-dependent effect of soybean feeding on the concentrations of daidzein, genistein, equol, and *para*-ethyl-phenol in the blood plasma of the control, early-pregnant, and late-pregnant heifers (adapted from [32]).

We have also used a cow model to compare metabolism of phytoestrogens in cyclic versus early-pregnant and late-pregnant heifers [32]. In this study, we found that in the blood plasma of the early- and late-pregnant heifers, there were lower concentrations of daidzein and genistein compared with control heifers at the mid luteal stage of the estrous cycle (Figure 3). In the blood plasma of the early-pregnant heifers, we noticed the decreases in isoflavone concentrations beginning at 3 h after soybean feeding, which was explained by acceleration of their metabolism leading to increases in the concentrations of their active metabolites, equol and *para*-ethyl-phenol [32] (Figure 3). In the late-pregnant heifers, we did not notice any increase in isoflavone metabolite concentrations after soybean feeding compared with the cyclic animals [32] (Figure 3). Taking other studies and above data into consideration, isoflavone absorption, biotransformation, metabolism, and bioavailability depend on various factors such as differences in digestive conditions, differences in the hormonal status of the animal during early and late pregnancy, and perhaps the most important factor, differences in immunological conditions connected with the phase of pregnancy [32, 34]. We also found out that during early pregnancy different isoflavone metabolism resulted from **β**-glucuronidase activation because of prompt changes in the immune system leading in turn to release of active forms of isoflavones into the blood plasma [32]. *β*-Glucuronidase is the enzyme responsible for isoflavone metabolism and biotransformation. It activates the release of free active forms of isoflavones from inactive conjugated with sulphuric and glucuronic acid forms. We have shown that isoflavone absorption and the concentrations of their metabolites in the blood plasma of late- or early-pregnant animals are completely different from those of animals during the estrous cycle [32] (Figure 3). Therefore, it could be assumed that there is some hormonal mechanisms that may lead to a decrease of soy-derived phytoestrogen absorption and deceleration of their metabolism, resulting in a lower active phytoestrogen metabolite concentration/accumulation in the blood plasma during late pregnancy in cows [32]. In fact, physiological status (cyclicity or pregnancy) of the female influenced the concentration/accumulation of isoflavone metabolites in the blood plasma of the heifers. Pregnancy had different effects on isoflavone absorption, biotransformation, and metabolism that resulted in higher concentrations of active metabolites of isoflavones during early pregnancy compared with lower concentrations during late pregnancy. Therefore, we surmised that early-pregnant heifers were more sensitive to hazardous active isoflavone metabolite actions than cyclic or late-pregnant heifers, and this in turn suggests that there are some other mechanisms preventing hazardous increases of the active metabolites of phytoestrogens in the blood plasma during late pregnancy [32]. Moreover, Kindahl et al. [35] documented that endogenous steroid metabolism changes during pregnancy due to various metabolic changes are connected with the conceptus. The data of Kindahl et al. [35] and our own [32] prove that exogenous estrogen metabolism changes during early pregnancy.

In humans, isoflavone absorption and bioavailability depend also on intestinal bacteria [36], gut transit time, fecal digestion rates, and the fiber content in the diet [37]. It has recently been reported in humans that within different physiological and pathological statuses, especially those connected with immune system mobilization, there is acute activation of **β**-glucuronidase activity leading to the release of active isoflavones into the blood plasma [38]. It has also been reported that this type of physiological immune system mobilization takes place during early pregnancy [34]. On the other hand, it has been known for a long time that the immune signals related to new embryo development are not only local, but spread very quickly throughout the entire female organism [39].

We also were the first to use the cow model to study isoflavone absorption and the concentrations of their metabolites in the blood plasma of the cows with the inflammation (experimentally induced *mastitis *and *metritis*) in comparison to healthy animals [40]. We found that the decrease in genistein concentration in the blood plasma of the cows with experimentally induced *metritis*, can be explained by acceleration of its metabolism leading to an increase in the concentration of its active metabolite, *para*-ethyl-phenol [40]. Kowalczyk-Zieba et al. [40] also documented higher *β*-glucuronidase activation during experimentally induced *metritis* connected with different isoflavone metabolism. Thus, the metabolism of isoflavones derived from the soybean (daidzein and genistein) was slower in the control and *mastitis *groups of cows compared to the cows with induced *metritis *[40]. The authors explained higher equol and *para*-ethyl-phenol concentrations in the blood plasma of cows with induced *mastitis* compared to control group due to the slight increase of *β*-glucuronidase activity in these cows compared to control animals [40]. Thus, during experimentally induced inflammations—*mastitis *or *metritis, *there is higher concentration of free, unconjugated phytoestrogen metabolites which may in turn influence on the immune system. In conclusion, Kowalczyk-Zieba et al. [40] found that *mastitis* and *metritis* in the cows influenced the accumulation of isoflavone metabolites in the blood plasma. Therefore, the authors suggested that cows with induced *mastitis* and *metritis* were more exposed to active isoflavone metabolite actions than healthy cows. We expected that during such inflammatory processes phytoestrogens can easier disturb reproductive processes including, modulation the hypothalamic-pituitary-ovarian axis or inhibition of gonadotropin secretion and [41, 42]. This caused a decrease of progesterone production which in turn led to high abortion rate [43]. Moreover, we hypothesized that at the time of *mastitis *and *metritis* phytoestrogens may disturb estrous and ovulation through their effects on the central nervous system [40].

### 3.2. Phytoestrogen Exposure Influences Reproductive Performance on Various Regulatory Levels

Phytoestrogens can disturb reproductive processes on different regulatory levels [44]. Many studies have been conducted on a ruminant model to define the direct effect of phytoestrogens within the central nervous system (CNS; pituitary gland and hypothalamus). Mathieson and Kitts [45] studied the binding of phytoestrogens to the estradiol receptor in the pituitary gland and hypothalamus. These authors indicated that phytoestrogens could interfere with the estradiol feedback mechanism to release luteinizing hormone (LH) in the ewe [45]. However, the effect of dietary exposure to phytoestrogens on LH secretion seemed to be dependent on the type of phytoestrogen and reproductive status and seasonality. In ovariectomized ewes, an increased concentration of coumestrol in the diet significantly reduced the amplitude of LH pulses during the breeding, but not during the anestrous season [46]. Furthermore, Romanowicz et al. [47] investigated whether genistein was capable of evoking effective changes in LH and prolactin (PRL) secretion in ovariectomized ewes during seasonal anoestrus. After several hours of genistein infusion into the third ventricle, plasma LH concentrations and the frequency of LH pulses decreased. Moreover, plasma PRL concentrations during and after genistein infusion were also significantly higher than the control. These data demonstrated that genistein may effectively modulate LH and PRL secretion in ovariectomized ewes by acting within the CNS [47].

Polkowska et al. [48] found that genistein infused to the third ventricle of the brain changed the endocrine activity strictly of LH-producing cells in the pituitary glands of ewes during the anoestral season. However, the infusion of genistein did not affect the expression of genes encoding FSH*β* and the storage of the *β*-subunit in the FSH-producing cells. The authors observed that genistein decreased the percentage and density of immunoreactivity of the LH*β*-positive cells, nevertheless stimulated the percentage and integral density of LH*β* mRNA-expressing cells. Furthermore, the increase in LH*β* mRNA in LH-positive cells of the treated animals was accompanied by an increased expression of ER*α* after genistein infusion. These results suggest that probably a rapid release of the hormone together with an enhanced synthesis of LH is possibly mediated by ER*α*. Data obtained by Polkowska et al. [48] implicated that genistein stimulated the expression of ER*α* in the LH*β*-expressing cells, decreased the pool of secretory granules stored in the LH-producing cells, and augmented the synthesis of *β* subunit for LH. Misztal et al. [49] analysed the effect of intracerebroventricularly genistein administration on growth hormone (GH) secretion in ewes. During the genistein infusion into the third ventricle of the brain, GH plasma concentration increased. Furthermore, several hours later, with the immunohistochemistry method the cited authors observed measurable diminished storage of GH in the pituitary somatotropes. The authors suggested that this plant-derived isoflavone, as 17*β*-estradiol [50], can be a stimulator of GH secretion in ewes and may exert its effect at the level of the CNS.

**Figure 4:**
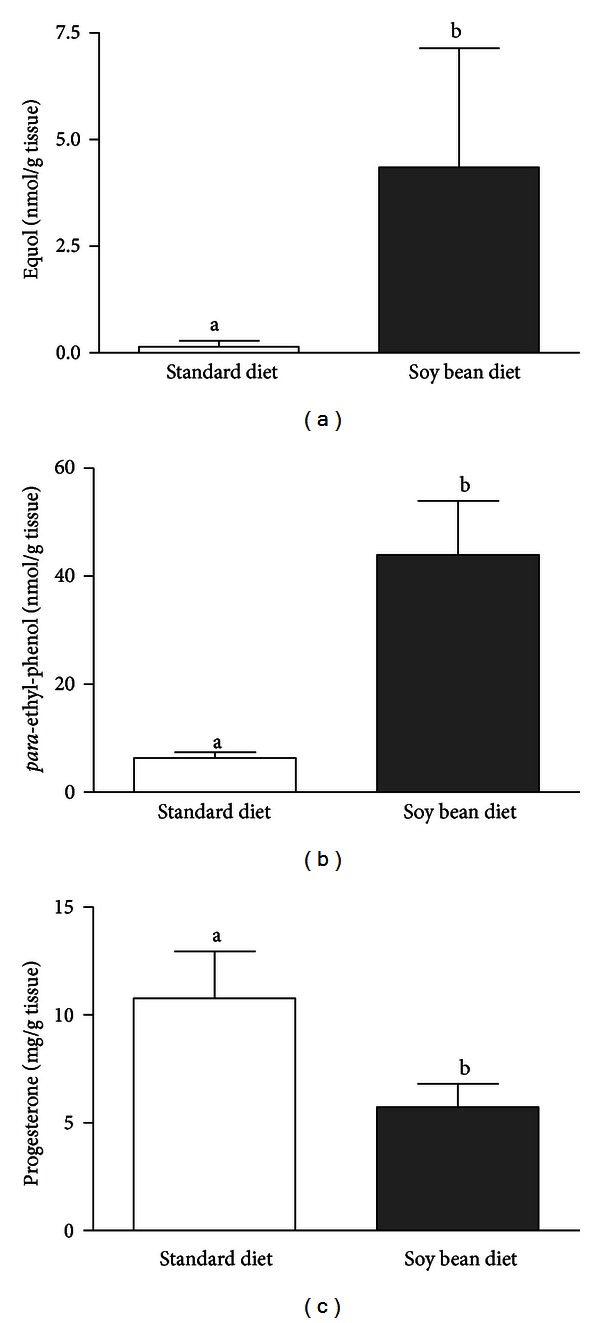
Concentrations of equol (a), *para*-ethyl-phenol (b), and progesterone (c) in the corpus luteum tissue of cows fed with soy diet (grey bars; 2.5 kg soy bean/animal/day) or with standard diet (white bars) (adapted from Piotrowska et al., 2008).

**Figure 5:**
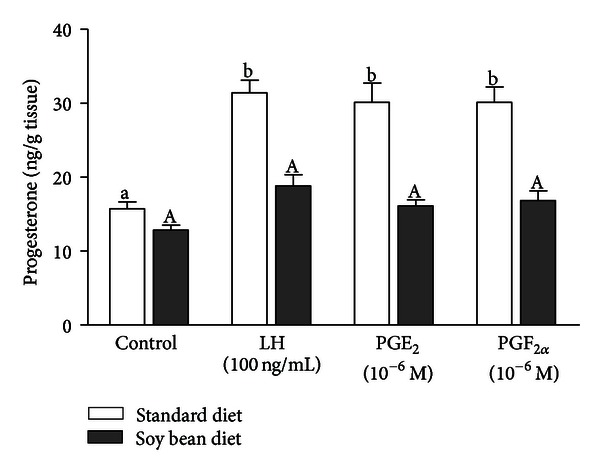
The effect of soybean diet on the LH-, PGE_2_-, and PGF_2*α*_-stimulated *in vitro* progesterone secretion by the bovine CL (adapted from Piotrowska et al., 2008).

**Figure 6:**
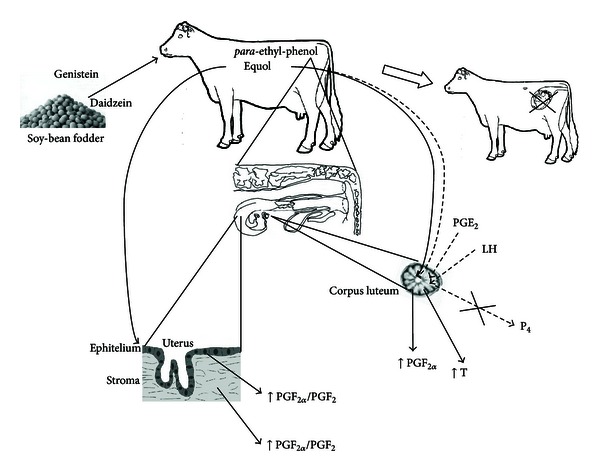
Possible influence of phytoestrogen action in the cow (the abbreviations on the figure stand for: LH—luteinizing hormone; P_4_—progesterone; PGE_2_—prostaglandin E_2_; PGF_2*α*_—prostaglandin F_2*α*_; T—testosterone).

The decrease of fertility can also be attributed to the local—direct effect of phytoestrogens on reproductive tract. Phytoestrogens can inhibit endogenous estrogen production in the ovary leading to disturbances in immune system regulation as well as in follicle development and lack of estrous [14]. High concentrations of active metabolites of phytoestrogens have been found in the CL tissues collected from heifers receiving soy diet compared to animals fed with standard fodder [42] (Figure 4). These high concentrations of phytoestrogen metabolites in heifers were associated with lower concentrations of P_4_ compared to heifers fed standard diet [42] (Figure 4). The authors of this study suggested that high concentrations of active metabolites of phytoestrogens present in the CL, directly disrupt its function by inhibiting P_4_ secretion [42]. Corpus luteum produces P_4_ required for the establishment and maintenance of pregnancy [51]. Therefore, active metabolites of phytoestrogens inhibiting P_4_ secretion may disrupt CL function and induce various disturbances during early pregnancy including the early embryo mortality [52]. On the other hand, it has been documented before that pituitary LH and luteal and/or ovarian PGE_2_ stimulate P_4_ production and output from bovine CL [53]. Piotrowska et al. [42] documented that LH and PGE_2_ stimulated P_4_ secretion in CL tissues collected from cows fed with standard diet in contrast to cows fed with soybean diet (Figure 5). These authors also found that in microdialyzed *in vitro* CLs, equol and *para*-ethyl-phenol inhibited LH-stimulated P_4_ secretion in comparison to the saline treated group. However, active metabolites of phytoestrogens did not influence basal P_4_ production *in vitro* [42]. Additionally, the experiments conducted on the bovine steroidogenic CL cells isolated from the late-luteal phase of the estrous cycle demonstrated that active phytoestrogen metabolites stimulated only luteolytic substance production—PGF_2*α*_ and T in the cells [54]. It was well documented before that in the cow, P4 is the main luteotropic hormone of CL origin [51], whereas PGF_2*α*_, E_2_, and T are the primary factors responsible for cessation of luteal P4 production and steroidogenic cell involution [55]. Therefore, any phytoestrogen-dependent increase in the PGF_2*α*_ secretion, and consequently elevation of E_2_ and T production at the late luteal phase, may lead to the termination of CL function and even abortion in case of early pregnancy [56]. Phytoestrogen-dependent stimulation of luteolytic PGF_2*α*_ and T in the steroidogenic CL cells at the luteal phase of the estrous cycle [54] agree with our previous *in vivo *studies, which proved that high soy diet significantly increased PGFM concentration in the serum of soy-fed animals causing the decrease of the rate of successful pregnancies and the increase of the mean insemination rate [31]. The influence of phytoestrogens and their active metabolites on P_4_ secretion is indirect, since it depends on the ability of phytoestrogens to inhibit LH and PGE_2_-stimulated P_4_ production. Feeding cows with high soybean diet may be the reason for disorders in the estrous cycle and several ovarian dysfunction during early pregnancy [31, 42, 54] (Figure 6).

In the series of *in vitro* experiments, we also studied local effects of phytoestrogens on the secretory function of the bovine endometrium [31, 57–59]. In these *in vitro* experiments, phytoestrogen metabolites (equol and *p*-ethyl-phenol) turned out to be much more potent disruptors than the original phytoestrogens themselves. We found that the stronger effects of the metabolites were due to their higher affinities for estrogen receptors than original phytoestrogens [31, 57, 58]. This hypothesis is supported by findings of other authors [44, 60] who showed that phytoestrogen metabolites are about 100–150% more active than environmental estrogens. We studied the influence of phytoestrogens derived from soybean and their metabolites on PGF_2*α*_ and PGE_2_ production in the cultured bovine endometrium at different stages of the estrous cycle [31]. Prostaglandins E_2_ and PGF_2*α*_ are crucial for proper development and maintenance of the CL. On the other hand, the maintenance of CL and P4 production is regulated by several luteotropic factors, including PGE_2_ [60]. However, the most important for the maternal recognition of pregnancy, maintaining the function of CL, embryo implantation and development is proper PGF_2*α*_/PGE_2_ ratio [56, 61] (Figure 6). Phytoestrogens and their metabolites greatly increased PGF_2*α*_ production and moderately but significantly increased PGE_2_ production during the luteal phase of the estrous cycle [31]. In case of pregnancy establishment, the PGF_2*α*_/PGE_2_ ratio should decrease. This relaxes the blood vessels and increases blood flow in the uterus, which prepares it for the embryo implantation [62]. The decreased PGF_2*α*_/PGE_2_ ratio also stimulates P4 synthesis [63]. Soybean phytoestrogens preferentially stimulated PGF_2*α*_ during the luteal phase of the estrous cycle (Wocławek-Potocka et al. [31]). Because PGF_2*α*_ has a direct and negative effect on bovine embryo development *in vitro* [64], the strong stimulation of PGF_2*α*_ production compared to PGE_2_ production that was observed in the bovine endometrial tissue may be one of the reasons of the early embryo mortality or abortion [31] (Figure 6).

However, when animals are not pregnant, during the estrous cycle (especially during late luteal and follicular phase of the cycle), this preferential PGF_2*α*_ stimulation can have positive effects on mechanisms responsible for luteolysis and returning the animals to cyclicity and ovulation [31]. During luteolysis, stimulation of PGF_2*α*_ secretion by estrogenic-like substances accelerates the positive feedback loop between PGF_2*α*_ and other regulators of luteolysis, such as, for example, oxytocin (OT) [56, 65] or TNF*α* [66, 67]. It was proved before that E_2_ increases OT-stimulated PGF_2*α*_ production in cultured bovine endometrial cells [68], as well as amplifies the stimulatory effect of OT on endometrial PGF_2*α*_ synthesis [69]. Additionally, gonadal steroids upregulate OT gene expression in the hypothalamus and upregulate OT receptors in the uterus; thus, they can alter the frequency of the central OT pulse generator, leading to the pulsatile PGF_2*α*_ output from the endometrium during luteolysis in ruminants [61, 70]. Therefore, the data obtained by Wocławek-Potocka et al. [31] proves that in this case if phytoestrogens and their metabolites act like endogenous estrogens, at the time of luteolysis and ovulation, they may amplify the mechanisms that return the cow to cyclicity after labor.

### 3.3. Intracellular and Enzymatic Mechanisms of Phytoestrogen Actions in Reproductive Tract

There are even more obstacles to overcome to study the intracellular and enzymatic mechanisms of phytoestrogen actions. The cow is also a relevant model for such kind of studies. Phytoestrogens and their metabolites differentially modulate PG synthesis in a cell-specific manner, increasing both PG without altering PGF_2*α*_/PGE_2_ ratio in stromal cells and directing the biosynthetic pathway toward PGF_2*α*_ in epithelial cells via stimulation of PGFS expression [31, 58].

It has been documented before that phytoestrogens inhibited the binding of (H^3^)-E_2_ or (H^3^)-Organon to their respective receptors, but the relative affinities of (H^3^)-E_2_ and (H^3^)-Organon were lower than those of E_2_ [14, 18, 71]. The affinities of phytoestrogens for estrogen receptors are only 0.1% to 1% of those of circulating estrogens (E_2_ or estrone) both in humans and cows [72]. Thus, the many biological effects attributed to phytoestrogens may be due to their relatively high concentrations. We found more than a thousand times greater concentrations of *p*-ethyl-phenol and equol (1.6 ± 0.31 *μ*M and 1.2 ± 0.28 *μ*M, resp.) in plasma of cows fed with soybean [31] than the concentrations of endogenous E_2_ (1–10 nM) [73]. These high concentrations may compensate much weaker affinity of phytoestrogens for estrogen receptors [18]. It has been previously shown that the concentrations of phytoestrogens in plasma of pregnant women consuming soybeans are over 1000 times higher than E_2_ concentrations and 10000 to 100000 higher than E_2_ concentrations during the menstrual cycle [9, 72, 74].

As mentioned before, estrogens in target tissues and cells exert their physiological effects by genomic [75] and nongenomic pathways [76] (Figure 2). However, we documented that phytoestrogens stimulate both PGF_2*α*_ and PGE_2_ in epithelial and stromal cells of bovine endometrium as well as PGF_2*α*_ production in the steroidogenic CL cells via only an estrogen-receptor-dependent, genomic pathway [31, 54]. Phytoestrogens and their active metabolites may compete with endogenous E_2_, thus disturbing the processes influenced by E_2_.

In the nongenomic pathway of estrogen action, PKA and PLC are the most important compounds of the intracellular second messenger system. Dubey et al. [77] found that genistein inhibited MAP kinase activity and PLD activity [78] as well as PLC-dependent intracellular calcium release [79]. However, in our previous study, neither the PKA inhibitor nor the PLC inhibitor (inhibitors of nongenomic pathways and second messengers), inhibited equol- and *para*-ethyl-phenol-mediated stimulation of PGF_2*α*_ synthesis in epithelial and stromal cells [57] or PGF_2*α*_ production in the steroidogenic CL [54], suggesting the lack of nongenomic mechanism of phytoestrogen metabolites action on the PG synthesis in bovine endometrium and CL, in contrast to endogenous E_2_.

Diverse effects on phytoestrogens on reproductive processes may depend not only on different intracellular and receptors pathways activation, but also on activation of various enzymes involved in arachidonic acid metabolism [58]. Although, phytoestrogens did not affect on either gene expression or protein level of prostaglandin-endoperoxide synthase-2 (PTGS-2; COX-2) and PGE_2_ synthase (PGES) in bovine endometrial stromal and epithelial cells, the stimulative effects of equol and *para*-ethyl-phenol on PGF_2*α*_ synthase-like 2 (PGFSL2) gene expression and protein level were observed in epithelial cells [58]. These results explain on enzymatic level why phytoestrogens can increase ratio of luteolytic PGF_2*α*_ to luteotropic PGE_2_ in bovine uterus [58] (Figure 6). The effect of estrogens and phytoestrogens on the viability of various types of cells was also studied in the literature. Phytoestrogens and their metabolites decreased the viability of bovine endometrial epithelial and stromal cells [58]. Similarly, Asselin et al. [68] and Nilsson et al. [80] also demonstrated that endogenous estrogens inhibit proliferation of epithelial cells and vascular endothelial cells in several organs. On the other hand, estrogens have been also reported to stimulate epithelial and endothelial cell growth and proliferation in the female reproductive tract of many animal species [81].

## 4. **Relevance of a Cow Model to Human Reproductive Performance**

Perfectly designed studies to examine the effects of isoflavones on humans should be done in human subjects. However, this situation is very hard to be accomplished. We have to take into account that in that kind of studies there are a lot of obstacles to overcome. Citing the group of Verkasalo et al. [82], there is usually wide variation in human exposures, these exposures are difficult to measure accurately, and the exposures are inherently difficult to control effectively. There is also extensive variability in isoflavone content of many dietary sources over time, whether standard food products or commercial botanical extracts are sold as dietary supplements [83]. What is more, the metabolism of isoflavones is not the same in all humans since there is different activity of metabolizing enzymes and also varies the influence of gut microflora on phytoestrogen bioavailability [84]. Summarizing, there are a lot of complications in the design and interpretation of human studies, combined with the ethical issues regarding experimentation in humans, that continuously increases interest in studies that utilize animal models. The relevance to human health of studies performed in animal models has been questioned many times in the literature, since in many of the animal studies exposure to phytoestrogens was by a nonoral route, whereas most human phytoestrogen exposure is from dietary intake [82]. This kind of exposures is usually chosen for rodent models of phytoestrogen exposure. Taking above arguments into consideration, it has been well documented that the cow can be a relevant animal model for studies of human reproduction because ovarian physiology and many aspects of embryo development, pregnancy and assisted reproductive techniques are similar between these two single-ovulating species [85, 86]. This model has broad applicability and may be used to extend investigations to different physiologic/pathologic states and to other species including humans. Moreover, the bovine model has the potential to be used as a sensitive *in vivo* bioassay to study the influences of xenoestrogens factors, including phytoestrogens on reproductive performance because of similar basic phytoestrogen metabolisms (genistein and daidzein) in both species (Figure 1).

Therefore, we believe that a cow model is far better since the main, natural exposure in this animal is also oral that does not vary from human exposure. The bovine model ensures a greater availability of biological material compared to studies in human. More importantly, the possibility to conduct *in vivo* studies represents a powerful tool that could possibly clarify the conflicting data obtained in different human studies. Altogether, these arguments support the use of studies in the cow in modeling exposure of humans to phytoestrogens.

## 5. **Adverse Effects of Isoflavones on the Reproductive Performance in Human**

The most common plant-derived proteins belong to soybean-based products. Isoflavones commonly enter the human body through the food chain. As the Oriental diet contains many soy-based products, isoflavone levels are high in the blood plasma of people living in the Oriental countries [87]. However, isoflavones are becoming more and more common in Western countries as well. This situation results from the increasing presence on the market of soy-derived dietary supplements, that represent ergonergic products for sportive people [88]. As a result, an increasing number of people in reproductive age assumes these phytoestrogens. Although these products are perceived as by the consumers “safe” because of being “natural”, in fact there is limited control on their safety [88].

Even though isoflavones are metabolized and excreted quite rapidly, their effects on human health can be remarkable. There are contradictory data in the literature on the isoflavone effect on human health. In this aspect, both beneficial and adverse effects of these natural estrogens are reported. Isoflavones, such as genistein and daidzein, have been addressed as preventive factors for cancer risk and cardiovascular diseases, and as antiobesity, neuroprotective, and osteoprotective agents [87, 89–91]. However, data on phytoestrogen action of estrogen sensitive tumors are contradictory [91, 92]. On one hand, epidemiological studies encounter a reduction in cancer incidence in populations consuming a soy-based diet, and on the other hand, some *in vitro* studies reveal some contradictory data [93–95]. It has been shown that phytoestrogens such as genistein, daidzein, and equol are able to mediate the proliferation of breast cancer cell lines [96, 97]. In particular, the modulation exerted by isoflavones on cancer cell lines seems dose dependent, with some doses promoting and other doses diminishing cell proliferation [98, 99].

Discussing diverse effects of phytoestrogens on human health differences and similarities about isoflavone metabolism in humans and ruminants should be taken into account. Similar to the cow, in some humans, daidzein—the main soy-derived isoflavone, can be transformed to equol by the intestinal flora [30, 100]. This metabolite is more bioactive than its parental compound in both human and other animals [10, 31, 101]. However, unlike ruminants, not all humans are able to produce equol. The ability to convert daidzein into equol derives from the different intestinal floras [102]. As equol shows much higher estrogenicity than its parent compounds, the effects exerted by isoflavones on human health should be more remarkable in “equol producers.” On the other hand, it has been demonstrated that, upon long-term exposure to isoflavones, “nonequol producers” can develop the ability to metabolize equol [103]. Thus, the differentiation between “equol producers” and “nonequol producers” depends mostly on the type of diet, and not on constitutive differences between individuals.

Another explanation for these contrasting data resides in the time frame in which the phytoestrogens exposure takes place, being the developmental window (i.e., pre- and early postnatal exposure), one of the most sensitive periods of human life. In fact, a big concern is arising from the use of soy-based infant formulas because of the delicate life period in which they are administered [104]. The exposure to phytoestrogens during prenatal and early postnatal life represents a matter of concern. Prenatal exposure can occur due to the life style of the mothers (e.g., vegetarian diet, dietary supplements intake, and soy milk intake) [87, 104]. Postnatal exposure often occurs because of soy-based infant formulas and soy milk intake.

Isoflavones cross the placental barrier and reach the fetal circulation [105, 106]. Many animal models have been applied for the study of intrauterine and perinatal exposure to hormones mimicking compounds of plant origin [107–111]. These studies demonstrated how intrauterine exposure to isoflavones can have consequences on the reproductive system in adulthood [108, 112, 113]. Unlikely for the exposure in adult life, the exposure in pre- or perinatal life seems to lead to irreversible alterations of the reproductive system. Such an effect might be due to epigenetic modifications that persist though the rest of life [113–115]. Male children exposed to isoflavones in utero showed hypospadias [116]. In this perspective, isoflavones can be encountered within the contributors, together with other hormone-mimicking compounds, to the decreasing efficiency in male reproduction registered in the last decades [8, 117]. Even though not registering significant differences, a study on infants fed with soy-based infant formulas appears worth of mention [118]. This study evaluated the differences in hormone-sensitive organs in infants fed with soy-based, milk infant formula, or with breast milk. Interestingly, a trend towards diminished testicular development was found in infants fed with soy-based or milk formulas. Exposure to genistein altered the male reproductive features not only in human [88, 119–122] but also in animal models [112, 123] and is not reviewed in this paper.

In utero exposure to isoflavones can also impair the reproductive system of female descendants. The evidence of such interferences comes mainly from animal studies. Isoflavones exposure in the womb resulted in a decreased sensitivity to the estrogen by the mammary gland [124]. If such finding reveals a possible cancer-preventive activity of isoflavones, on the other hand it raises concern for other possible health outcomes. In particular, isoflavones exposure during fetal life alters the estrogen receptor ratios, thus impairing the physiological action of estrogens. Surprisingly, genistein administration during fetal life resulted in an increased risk of uterine cancer and in a promotion of leiomyoma [125]. Perinatal exposure to isoflavones resulted in alterations in the uterus and ovaries of female pups [126, 127].

On the other hand, consumption of isoflavones in women reproductive age has been linked to dysmenorrhea, endometriosis, and secondary infertility [16, 128]. A high intake of phytoestrogens resulted in dysmenorrhea and persistent sex arousal syndrome in one case-study reported by Amsterdam et al. [15]. In this study, like in the one reported by Chandrareddy et al. [16], withdrawal of soy intake from the diet resulted in the lessening or in the complete disappearance of the symptoms. Remarkably, in the studies of Chandrareddy et al. [16] one patient was able to conceive after isoflavone withdrawal from her diet. Keeping in mind that these adverse effects have been encountered only in a restricted number of cases, it still appears advisable to handle the phytoestrogens' intake with care. *In vitro* studies strengthen the observation that isoflavones can directly modulate endometrial physiology [99, 129]. Interestingly, genistein was able to modulate the proliferation of Ishikawa cells, an epithelial cell line derived from adenocarcinoma, in a dose-dependent fashion, being the low doses an inhibitor factor for proliferation, that was instead promoted by high doses [99]. At similar doses, genistein promoted the proliferation of leiomyoma cells [129]. These findings raise concern for the beneficial effects of isoflavones. Surprisingly, genistein revealed to cure endometrial hyperplasia in a clinical trial [130].

Isoflavones can exert their effect not only on the uterus level. Other estrogen-sensitive organs such as ovaries can be affected by these natural estrogens. In order to guarantee a normal ovarian function, estrogen circulating levels must oscillate during the cycle. Low estrogen levels stimulate FSH release by the hypothalamus/pituitary, thus leading to follicle growth. The presence of isoflavones can nullify the required low levels of endogenous estrogen. This could lead to irregular cycle, and even to reproductive impairment [131]. Following soy intake, cases of altered steroid hormones levels and trends for increased cycle length have been reported [131–133]. Moreover, soy supplementation to women in reproductive age resulted in decreased LH and FSH levels during the periovulatory phase [133]. If such alterations can be sufficient to impair the ovarian cycle is still argument of debate. Moreover, there are conflicting results on the effects of isoflavones on the hypothalamus-pituitary-gonads axis [132, 133]. Thus, it is not possible to evaluate if the effects exerted by isoflavones on human reproduction are due to a local or a systemic action. Interestingly, animal studies demonstrated that genistein is able to impair ovarian differentiation in mice [134, 135]. In this light, the results collected among women in reproductive age raise great concern for the effects of isoflavones' exposure [16, 134]. Fortunately, the effects exerted by isoflavones in adult life appear reversible once dietary intake is ceased [131].

Women in menopause represent another important category of people exposed to high concentrations of isoflavones [136, 137]. However, there are many reports showing that dietary supplements containing genistein seem to lessen menopausal symptoms [137]. While phytoestrogens seem to exert a positive effect on postmenopausal women, their effect could be deleterious in women in reproductive age. Isoflavones lessen menopausal symptoms and do not seem to show the contraindications of the estrogen replacement therapy, even though some exceptions have been registered [136–140]. In particular, genistein is able to promote estrogen synthesis in an extragonadal pathway, thus exerting a positive effect in menopausal women [141].

To summarize, the data reported above clearly indicate that phytoestrogens are able to modulate important processes of human physiology. The conflicting results encountered in the literature do not allow us to draw conclusions on whether phytoestrogens exert a positive or a negative effect on human reproductive health. The often opposite effects registered in the available literature can be generated by the different genders, ethnics, and, more importantly, at different time-frame of exposure considered. Thus, the effect of isoflavones on reproductive efficiency in humans should be investigated on a relevant animal model.

## 6. **Conclusions**

There is overwhelming evidence in many studies using a ruminant model that phytoestrogen exposure can have significant consequences for reproductive health. The effects of phytoestrogens depend on many various conditions such as dose and route of exposure because these parameters impact the final serum level of the bioactive compound. Moreover, the timing of exposure is critical in determining the phytoestrogen-induced effects and different tissues have species-specific windows of sensitivity to morphological and functional disruption. However, the most important issue connected with phytoestrogens is the fact that they are more and more commonly recognized as therapeutic compounds. Therefore, it is crucial to examine carefully the effects of these chemicals on reproductive outcomes using animal models that replicate human exposure levels.

In spite of many limitations in conducting well-designed human studies, information gathered from already published ones combined with the large number of animal studies already available, clearly demonstrate that phytoestrogens have the ability to influence the reproductive performance of an adult. These findings should be specially taken into consideration when recommendations are made regarding dietary or therapeutic phytoestrogen intake in humans.
